# Wear Comfort Characteristics of Al_2_O_3_/ATO/TiO_2_-Embedded Multi-Functional PET Fabrics

**DOI:** 10.3390/ma15248799

**Published:** 2022-12-09

**Authors:** Hyun-Ah Kim

**Affiliations:** Korea Research Institute for Fashion Industry, 45–26, Palgong-ro, Dong-gu, Daegu 41028, Republic of Korea; ktufl@naver.com

**Keywords:** Al_2_O_3_/ATO-embedded yarn, wear comfort, moisture absorption and fast drying, heat retention rate, moisture vapor resistance

## Abstract

The wear comfort of Al_2_O_3_/ATO (antimony tin oxide, Sb_2_SnO_5_)/TiO_2_-embedded fabrics was examined in terms of the heat shielding property of ATO particles and emissivity of far-infrared (FIR) radiation according to the sheath/core ratio (wt. %) of Al_2_O_3_ and ATO particles embedded in the sheath/core yarns. The thermal radiation of the low-core-ratio (50 wt. %) Al_2_O_3_/ATO-embedded fabric was greater than that of the high-core-ratio (70 wt. %) Al_2_O_3_/ATO-embedded fabric, which was verified by the higher FIR emissivity. The heat-shielding effect of the ATO particles in the highly Al_2_O_3_/ATO-embedded yarns enabled it to shield the FIR emitted from sunlight, resulting in lower emissivity and emissive power of the highly embedded Al_2_O_3_/ATO yarn than lower embedded yarn. Accordingly, lower Al_2_O_3_/ATO-embedded yarns are required to obtain excellent thermal radiation of the fabrics. The fabric with a low core ratio (50 wt. %) exhibited superior moisture absorption, fast-drying properties, and a higher heat retention rate than fabrics with a high core ratio (70 wt. %). Furthermore, the fabric with a low core ratio exhibited superior breathability to the fabric with a high core ratio, resulting in easy moisture vapor permeability. These findings show that the fabric with a low core ratio (50%) of Al_2_O_3_/ATO particles imparts superior absorption, moisture vapor permeability, and thermal wear comfort to the high-core-ratio fabric (70%), giving it a comfortable wear-comfort feel while wearing the clothing. Hence, low-core-ratio yarn is suitable for warm-up fabric with UV-protection and anti-static characteristics.

## 1. Introduction

For many years, infrared radiation has been used for drying in the textile industry, and related research has been carried out [1,2,3,4]. Recently, studies on the interactions between the heat release and storage characteristics of ceramic-embedded fabric and far-infrared radiation and its absorbing properties have attracted increasing attention [5,6,7,8,9]. Ceramic particles exhibit heat release characteristics when exposed to sunlight and can be divided into three types of materials: ZrC, CNT (carbon nano-tube), and graphite as carbon compounds; TiO_2_, ZnO, ATO (antimony tin oxide, Sb_2_SnO_5_), and Al_2_O_3_ as metal oxide compounds; and SiO_2_ as a nonmetallic compound. Unitica Ltd.Co. and Descente Ltd. Co. in Japan developed a solar-energy-absorbing and a heat-retaining fabric called ‘Solar α’ using a ZrC-embedded filament [10]. Studies using TiO_2_ [6,7], Al_2_O_3_, and SiO_2_ [8,9] have been reported. These studies examined the heat storage and release properties with the infrared emission of PET (polyethylene terephthalate) yarns and their fabrics embedded by these various ceramic particles. The most popular studies were carried out using ZrC-embedded yarn and its fabric because of its high thermal radiation efficiency [10,11,12,13,14].

Lin et al. [15] reported the far-infrared emissivity of the master batches composed of polypropylene and various ceramic powders (Al_2_O_3_, Fe_2_O_3_, CaO, ZrO_2_, TiO_2_, and SiO_2_). Kuo et al. [16] reported the effectiveness of the polypropylene fibers mixed with SiO_2_ and ZnO in retaining heat with the emissive energy absorbed and converted by far-infrared (FIR) rays. In previous studies, various ceramic particles imparted heat-release characteristics to the yarns and fabrics. Moreover, yarns and fabrics embedded with different mixed ceramic particles showed increasing solar absorption and emission energy at the mid-infrared region and superior functional properties such as UV-protection and anti-static properties.

UV radiation harms the human body when human skin is exposed to sunlight. Many studies [17,18,19,20,21,22,23,24] were performed to improve UV protection using TiO_2_, ZnO, Al_2_O_3_, and SiO_2_ ceramic particles. Of these ceramic particles, TiO_2_ and ZnO were the most popular UV-cut agents, and they increased the effectiveness of UV protection by a nanoparticle-treated coating on the fabric surface. On the other hand, many studies [25,26,27,28,29,30] have been undertaken to improve the anti-static property of synthetic fibers using various ceramic particles. Prior studies [26,27] reported that TiO_2_, ZnO, and ATO particles have anti-static properties. In particular, ATO has excellent heat insulation and shielding (IR absorbing), good electric conductivity (anti-static), and environmentally friendly properties [17]. ATO particles help to effectively dissipate the static charge accumulated on the fabric [30]. According to the prior study [30], ATO particles have appropriate structures for high electric conductivity, i.e., the electric conductivity of ATO is dependent on the antimony and its oxidation state in the tin oxide lattice. Electrical resistivity is decided by the ratio of Sb^5+^ and Sb^3+^ sites as both Sb^5+^ as electron donor and Sb^3+^ as electron acceptor coexist. This is the structural characteristic of ATO and the reason for higher electric conductivity of ATO ceramic particles. Ahn et al. [28] investigated the thermal insulation properties of ATO-coated glass materials applicable to the eco-friendly field. Sun et al. [29] studied the physical properties of the ATO-coated PET films with excellent electric conductivity applicable to the anti-static field. Muller et al. [30] synthesized nano-sized mono-dispersed antimony-doped tin oxide particles with highly conductive characteristics using a nonaqueous sol-gel method.

Previous studies conducted using a coating method had a weakness in not maintaining the multi-functional characteristics such as anti-static and UV-protection properties according to repeated washing and laundering while wearing clothing, i.e., a novel method for this, not a coating method, is required. Furthermore, few studies have investigated UV protection and anti-static characteristics with FIR radiation of the Al_2_O_3_-, ATO- and TiO_2_-embedded fabrics. In particular, multi-functional warm-up fabrics with UV-protection and anti-static properties are required during winter mountain climbing [31,32]. Recently, in a prior study [33], ATO-embedded sheath/core PET yarns were developed from a bi-component spinning method using an improved scheme. The UV-protection and anti-static properties of the ATO-embedded PET fabrics were examined and revealed the possible applicability of this fabric to multi-functional warm-up clothing. In addition, a prior study [33] reported that ATO particles embedded in the yarns provided superior anti-static properties to the Al_2_O_3_ and TiO_2_ particles, and the lower ATO (wt. %)-embedded fabric exhibited higher UV protection than the higher ATO-embedded one.

In general, clothing also requires superior absorption and moisture vapor permeability and thermal wear comfort during the wearing of the clothing. In addition, research on a Al_2_O_3_/ATO-embedded new fabric material conducted in prior study [33] may be a preliminary study on materials with protective properties. Moreover, further study for previous study [33] is required to examine wear comfort characteristics with protective properties of the Al_2_O_3_/ATO-embedded fabrics.

Therefore, based on a previous study [33], this study examined the wear comfort such as moisture absorption and drying properties, moisture vapor permeability (breathability), and thermal properties of the fabric specimens made from sheath/core PET yarns embedded with Al_2_O_3_/ATO/TiO_2_ particles.

## 2. Materials and Methods

### 2.1. Preparation of Yarn Specimens

Three Al_2_O_3_/ATO/TiO_2_-embedded PET filaments were spun on a conjugated melt spinning machine (TMT Co., Ltd., Kyoto, Japan) using Al_2_O_3_ and ATO master batch (M/B) chips. The Al_2_O_3_ and ATO M/B chips were prepared using a mixed polymer combined with 20 wt. % Al_2_O_3_ particles and 80 wt. % PET chips and with 20 wt. % ATO particles and 80 wt. % PET chips on a compounding machine (SM Platek Co., Ltd., Ansan, Republic of Korea), respectively. Table 1 lists the specifications of the master batch chips with their shapes and SEM images of the Al_2_O_3_ and ATO particles. The ceramic particles used in this study were filtered using a mesh of 600–800 nm for Al_2_O_3_ and 200–300 nm for ATO. The particle size distributions were assessed using hydro 2000S (Malvern Panalytical, Malvern, UK). The average size in Table 1 is the mean diameter measured using hydro 2000S.

Al_2_O_3_/ATO-embedded PET yarn was prepared using polymers mixed at a mixing ratio of 0.8 wt. % Al_2_O_3_ (2 kg) and 0.3 wt. % ATO (0.75 kg) master batch chips, which were combined with a TiO_2_-embedded PET base polymer (47.25 kg) in the core part of the conjugated melt spinning machine. Table 2 lists the mixing ratio of the master batch chips to make the Al_2_O_3_/ATO sheath/core PET yarn specimens.

**Figure 1:**
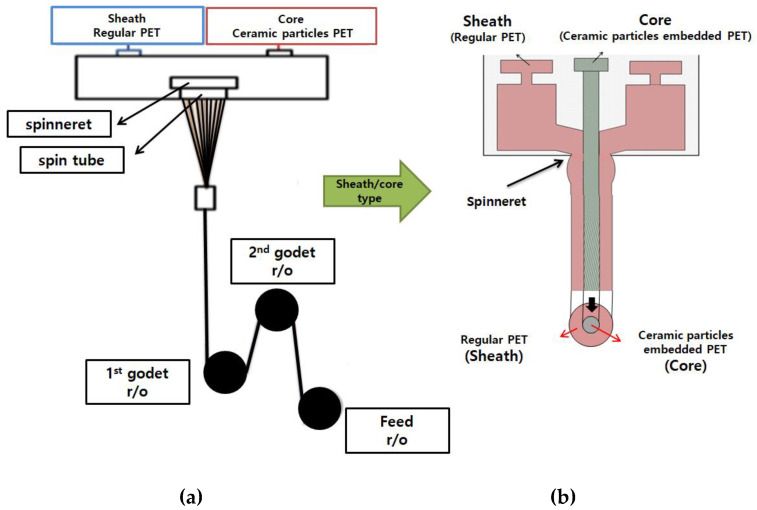
Schematic diagram of conjugated spinning machine ((**a**) conjugated spinning system and (**b**) bi-component spinneret).

Three types of Al_2_O_3_/ATO-embedded yarn specimens were made with the PET polymer (50, 40, and 30 wt. %) in the sheath part and the Al_2_O_3_/ATO-embedded PET polymer (50, 60, and 70 wt. %) in the core part on a conjugated spinning machine, resulting in different ratios (wt. %) of sheath/core yarn specimens (50/50, 40/60, and 30/70). In addition, yarn specimen 4 was prepared as a regular PET yarn (50d/72f), which was adopted by an existing yarn as a control yarn. These yarn specimens are listed in Table 2. The conjugated spinning system was equipped with a 24-hole spinneret with a capillary diameter of 0.24 mm and a length of 0.5 mm. Figure 1 presents a schematic diagram of the conjugated melt-spinning machine. The spinning temperature in the pack was changed from 280 to 290 °C to determine the optimal spinning conditions, and the spinbeam temperature was determined to be 287 °C. The heating temperature in the extruder ranged from 300 °C to 305 °C in the sheath and 301 °C to 304 °C in the core. The 1st godet roller speed and temperature were 1446 m/min and 85 °C, respectively. The 2nd godet roller speed and temperature were 3910 m/min and 105 °C, respectively, and the feed roller speed was 4000 m/min. The 50d/24f sheath/core PET filament was spun with a sheath/core wt. ratio of 5:5, 4:6, and 3:7.

### 2.2. Preparation of the Fabric Specimens

The fabric specimens were prepared on the water-jet loom (ZW-315X, Tsudakoma, Japan) using four types of weft yarn with a fixed warp yarn of 50d/72f PET filament: three types of Al_2_O_3_/ATO-embedded sheath/core yarn (sheath/core weight %: 5:5/4:6/3:7) and one regular PET yarn as a control yarn. Table 3 lists the specifications of the four types of fabric specimens. The fabric density was set at the state of grey fabric, and fabric thickness and weight were measured using a compression meter (FAST-1) and KSK 0514 after the finishing process. The weave structure was plain. The grey fabric specimens were scoured in a CPB scouring machine (BPB, Kuester, Germany) and washed and dried on a continuous drying machine (Extra-CTA 2400, Benninger, Switzerland). Dyeing was performed on a rapid dyeing machine (Cut-MF-1, Hisaka work, Ltd., Osaka, Japan) and finally washed, dried and followed by final setting in the stenter machine (Sun-super Ilsung Ltd. Co., Daegu, Republic of Korea).

### 2.3. FIR Analysis of the Yarn Specimens

**Figure 2:**
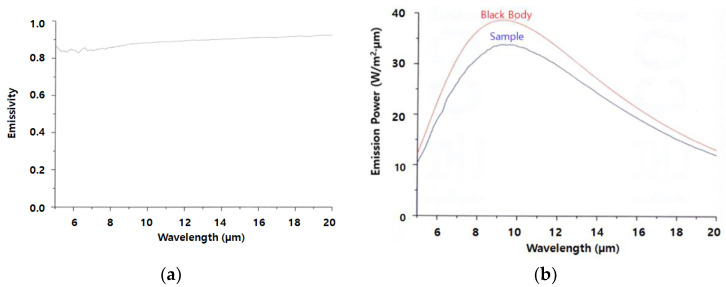
Emissivity and emissive power of the yarn specimen. ((**a**) emissivity and (**b**) emissive power).

Fourier transform infrared (FT-IR, Midac M 2400-C, Irvine, CA, USA) spectroscopy assessment was performed to estimate the FIR radiation from the ceramic particles embedded in the yarn specimens. The emissivity and emissive power of the yarn specimens were measured at 40 °C over the wavelength range of 5–20 µm using FT-IR spectroscopy. The means and deviation were calculated using five readings of experimental data and the unit of emissive power was W/m^2^∙μm. Figure 2 shows a diagram of emissivity and emissive power measured in this study. The emissivity shown in Figure 2a is defined as Equation (1).

(1) $$\mathrm{Emissivity}\;=\frac{\mathrm{sum}\;\mathrm{of}\;\mathrm{emissive}\;\mathrm{power}\;\mathrm{of}\;\mathrm{the}\;\mathrm{specimen}}{\mathrm{sum}\;\mathrm{of}\;\mathrm{emissive}\;\mathrm{power}\;\mathrm{of}\;\mathrm{the}\;\mathrm{balck}\;\mathrm{body}}$$

### 2.4. Thermal Radiation Measurement

A thermal radiation assessment was performed to measure the temperature rise by the heat emitted from the ceramic particles in the yarns and fabrics using a light heat emission apparatus [33]. A 10 × 10 cm specimen was prepared at 20 ± 2 °C and a relative humidity of 65 ± 5% and placed on a thermometer equipped with a contact-type sensor (PT100, Omega engineering, Stanford, CA, USA) in the specimen die. The heat emission bulb was placed 30 cm away from the specimen, which was switched on and off. The temperature change according to the measurement time was determined to detect the heat-release property by FIR radiation of the ceramic-embedded yarns and fabrics. Five independent replications of the test were conducted.

### 2.5. Moisture Absorption and Drying Properties Using the Moisture Management Tester (MMT)

**Figure 3:**
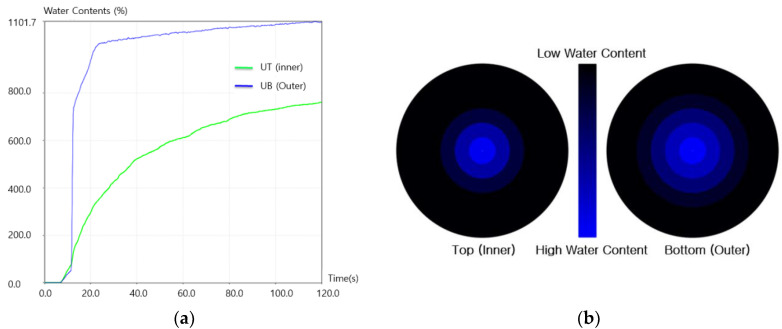
Diagram of the MMT experimental result ((**a**) moisture content vs. time and (**b**) moisture location vs. time).

The moisture absorption and drying properties of the fabrics are very critical to evaluating the wear comfort of the clothing. The moisture absorption of the four types of fabric specimen was measured using the MMT [34]. The test was carried out according to the standard AATCC (American Association of Textile Chemists and Colorists) test method 15 [35]. Five pieces of the four types of fabric specimen were cut into 80 × 80 mm squares and used as specimens in the MMT experiment. The measurement was performed by dividing it into two steps: locating the measuring places and determining what to wet before measuring. A special solution (0.15 g) mixed with distilled water and sodium chloride was injected automatically onto the top surface of each specimen to stimulate sweating. The wetting time (sec) of the fabric specimens was measured on the top and bottom surfaces, which is when the top and bottom surfaces of the fabric started to become wet. The absorption rate is measured and calculated using Equation (2), as shown in Figure 3a.

(2) $$\mathrm{Absorption}\;\mathrm{rate}\;=\;\mathrm{initial}\;\mathrm{slope}\;\left(\frac{\mathrm{water}\;\mathrm{content}\;\left(\%\right)}{\mathrm{time}\;\left(s\right)}\right)$$

Figure 3 presents the raw data measured in this study using an MMT. The MMT is designed to measure the liquid moisture transport behaviors in multiple direction. The radius of the circle in Figure 3b is defined by the distance between the center of the wetted ring and wetted circle. The maximum wetted radius (MWR, mm) is the radius of the circle, shown in Figure 3b, which was recorded automatically in this apparatus. Finally, the spreading speed (mm/s) was defined as the velocity from the center of the wetted ring to the MWR, which was also calculated automatically by MWR/t, where t is the time to reach the maximum wetted ring.

### 2.6. Measurement of the Thermal Conductivity and Heat Retention Rate

**Figure 4:**
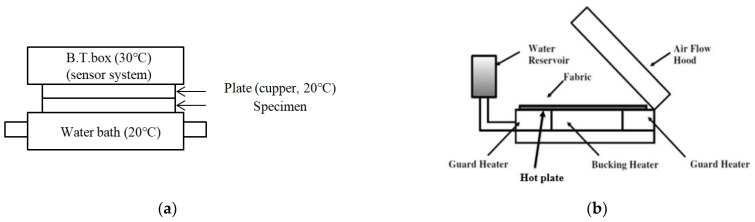
Schematic diagram of the measuring apparatus ((**a**) KES-F7 and (**b**) sweating-guarded hot plate) [36].

Understanding how the heat released from ceramic-embedded yarn and fabric influences the heat retention rate and the thermal conductivity is essential for examining the wear comfort of the clothing. The thermal conductivity (K) was measured using a KES-F7 (Kato Tech Co., Kyoto, Japan) at 20 ± 1 °C and 70 ± 5% RH. Figure 4a shows a schematic diagram of the KES-F7 apparatus [36]. The temperature in the B.T. box was set to 30 °C, and water in a water bath was circulated at 20 °C. A fabric specimen was placed in the water bath. Therefore, heat in the apparatus flows from the B.T. box (30 °C) to the water bath (20 °C) through a plate and specimen. The B.T. box then measures the heat loss (H) from the plate in watts (W) by the change in electrical voltage. The thermal conductivity (K) was calculated using Equation (3) to examine the dry heat transmission of the ceramic-embedded fabrics.

(3) $$K=H\;\cdot \frac{d}{A}\cdot \;\Delta T$$

where K, H, d, A, and ΔT are the thermal conductivity (W/°C), heat loss (W), fabric specimen thickness (cm), fabric specimen area (cm^2^), and temperature difference (°C), respectively. In addition, the heat retention rate (I) was measured and calculated using Equation (4).

(4) $$I=\left(1-\frac{b}{a}\right)\times 100$$

where a is the heat emanating from the test plate (W), and b is the heat emanating from the mounted fabric specimen (W).

### 2.7. Measurement of the Moisture Vapor Resistance

The moisture vapor resistance (breathability) in the clothing is very important for examining the wear comfort of the ceramic-embedded fabrics. The moisture vapor resistance (R_et_) of the fabric specimens related to the wet heat transport was measured using a sweating-guarded hot plate (Therm DAC, Daventry, UK) according to the ISO 11092 method [37]. Figure 4b shows a schematic diagram of a sweating-guarded hot plate apparatus [36]. A fabric specimen, 30 × 30 cm in size, was prepared and conditioned in a standard atmosphere (RH of 65%, 20 °C). The fabric specimen was placed over the breathable (PTFE) membrane covered with perforated metal on a hot plate, which prevents moisture on the perforated metal from wetting the fabric specimens [36]. The temperature of the guarded hot plate was kept at 35 °C, where the air speed is 1 m/s. The arithmetic mean of five readings from each fabric specimen was calculated. The moisture vapor resistance (R_et_) of the fabric specimen was determined by measuring the power (heat loss, H) under the steady-state condition and was calculated using Equation (5).

(5) $$R_{\mathrm{et}}=\frac{A\left(P_{a}-P_{m}\right)}{H-∆H_{e}}-R_{\mathrm{eto}}$$

where R_et_, p_m_, and p_a_ are the moisture vapor resistance (m^2^ Pa/W), saturated moisture vapor partial pressure (Pa) at the surface of the measuring unit, and moisture vapor partial pressure (Pa) of the air in the test, respectively. A, H, ΔH_e_, and R_eto_ are the surface area of the measured perforated plate (0.04 m^2^), power (W) required to maintain a constant plate surface temperature, correction power (W), and moisture vapor resistance (m^2^ Pa/W) of a bare plate, respectively.

## 3. Results and Discussion

### 3.1. Characteristics of the Al_2_O_3_/ATO/TiO_2_-Embedded Sheath/Core Yarns

**Figure 5:**
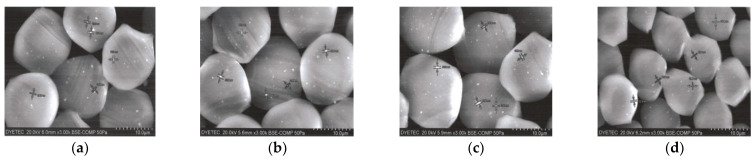

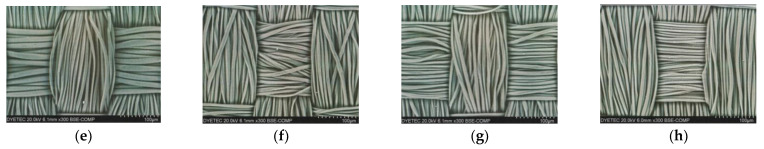
SEM images of the cross-section and surfaces of the four types of yarn specimens. (**a**) specimen 1, (**b**) specimen 2, (**c**) specimen 3, (**d**) specimen 4, (**e**) specimen 1, (**f**) specimen 2, (**g**) specimen 3, (**h**) specimen 4.

Knowing how the Al_2_O_3_/ATO particles are embedded in the yarns is very important for understanding the wear comfort and heat release of the ceramic-embedded fabrics. Figure 5 presents SEM images of the cross-sections and surfaces of the four types of yarn specimens. As shown in Figure 5a–d, many white spots in the yarn cross-section appeared, which were assumed to be ceramic particles such as Al_2_O_3_, ATO, TiO_2_.

Figure 5e–h show SEM images of yarn surfaces in the four fabric specimens. Small white spots on the yarn surfaces are shown, which are assumed to be TiO_2_ particles embedded in the sheath of the core/effect yarns. No significant difference among SEM images of the four yarn surfaces was observed in the specimens 1, 2, 3, and 4.

### 3.2. FIR Characteristics and Thermal Radiation of the Ceramic-Particle-Embedded Yarns and Fabrics

An investigation of how the thermal radiation and FIR characteristics of the ceramic-embedded fabrics are affected by the wt. % of Al_2_O_3_/ATO particles in the yarns is essential for understanding the wear comfort and heat release of ceramic-particle-embedded fabrics. Table 4 lists the emissivity and emissive power of the yarn specimens [33]. ANOVA (F-test) between the mean value of the four specimens was performed to verify the statistical significance with the 95% confidence limit. Table 5 lists the ANOVA results between the mean value of the emissivity and emissive power of each yarn specimen. As shown in Table 5, the mean value between each yarn specimen was significant as F_0_ (V/Ve) > F (3, 16, 0.95) and *p* < 0.05.

**Figure 6:**
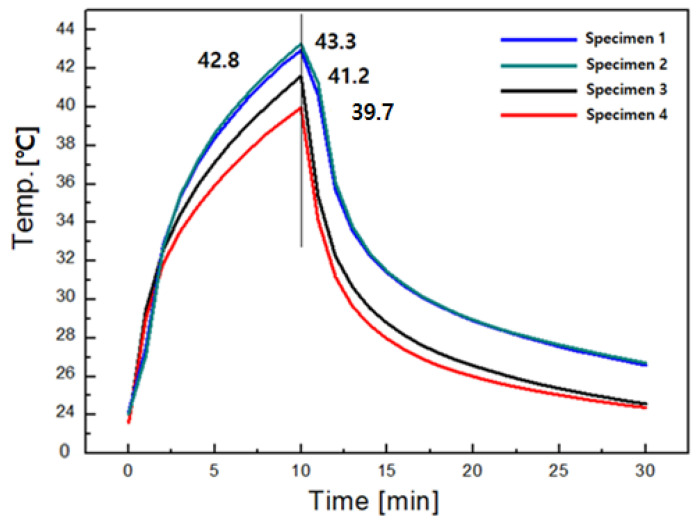
Thermal radiation diagram of the four types of fabric specimens.

The emissivity and emissive power of the Al_2_O_3_/ATO-embedded yarns (specimens 1, 2 and 3) were higher than those of the TiO_2_-embedded yarn (specimen 4), which indicates that Al_2_O_3_/ATO-embedded yarns have higher effectiveness for the FIR emission than TiO_2_-embedded yarn (Table 4). The higher emissivity (higher effectiveness) indicates more heat released from the ceramic particles embedded in the yarns, which was verified by the maximum surface temperature from the thermal radiation experiment, as shown in Figure 6 [33]. The maximum surface temperature of the Al_2_O_3_/ATO-embedded fabrics (specimens 1, 2 and 3) was higher than that of the TiO_2_-embedded fabric (specimen 4), which might be due to the greater amount of heat released from the Al_2_O_3_/ATO-embedded fabrics than the TiO_2_-embedded fabric. According to a previous study [16], FIR transmits heat energy in electromagnetic waves by radiation when the ceramic particles in the yarns are irradiated. In contrast, electromagnetic waves are likely to be absorbed by the human body for warming. Specifically, heat is released from the absorption or accumulation of the FIR emitted from the ceramic particles in the yarns exposed to light. Accordingly, more heat release of the Al_2_O_3_/ATO-embedded fabrics was attributed to the higher emissivity of FIR of these fabrics. Pooley et al. [6], Lin et al. [15], Lin and Chang [38], and Lin et al. [39,40] carried out similar attempts related to FIR emissivity. They measured the emissivity of the TiO_2_ particles and bamboo-charcoal-embedded materials, and the interaction between the heat released from the ceramic particles and FIR was examined. They reported the heat release characteristics due to the high FIR emissivity of the various ceramic particles embedded materials.

In addition, regarding the emissivity and emissive power according to the wt. % of the Al_2_O_3_/ATO particles embedded in the core among yarn specimens 1, 2, and 3, the emissivity and emissive power of yarn specimen 1 were higher than that of yarn specimens 2 and 3, as shown in Table 4. Specifically, the specimen with less ATO in the core of the yarns exhibited higher emissivity. This was verified by the maximum surface temperature shown in Figure 6, i.e., the maximum surface temperature of fabric specimen 1 (core (Al_2_O_3_/ATO) wt. %: 50) was higher than that of fabric specimens 2 and 3 (core (Al_2_O_3_/ATO) wt. %: 60 and 70). This was attributed to the heat-shielding properties of the ATO in the yarns. That is, the heat shielding property of the ATO decelerates the temperature rise from the heat radiated by FIR emanating from the ceramic particles embedded in the yarns, resulting in a lower maximum surface temperature of fabric specimen 3 with a high core ratio (70 wt. %) than fabric specimen 1 with a core ratio of 50 wt. %. This suggests that the lower core wt. % ratio (5:5) of the sheath/core in the Al_2_O_3_/ATO-embedded yarn (specimen 1) is more effective for heat release due to less heat shielding than the higher core wt. % ratio (3:7) of the sheath/core yarn (specimen 3).

In summarizing FIR and thermal radiation according to the wt. % of Al_2_O_3_/ATO particles embedded in the yarns, the heat shielding effect of the ATO particles enables it to shield the FIR emitted from sunlight, resulting in a lower emissivity and emissive power of the highly embedded ATO yarn (70 wt. %) than the lower embedded one (50 wt. %). This was verified by the lower maximum surface temperature of the highly embedded ATO fabric than the low embedded ATO fabric. Although the higher embedding of the Al_2_O_3_ particles in the highly embedded Al_2_O_3_/ATO fabrics enhances heat release due to the higher FIR emissivity, the heat shielding effect due to the higher ATO particles was much more dominant than the heat release of Al_2_O_3_. Accordingly, the lower Al_2_O_3_/ATO-included yarns are required to produce excellent thermal radiation fabrics while wearing clothing.

### 3.3. Moisture Absorption and Drying Properties by MMT

**Figure 7:**
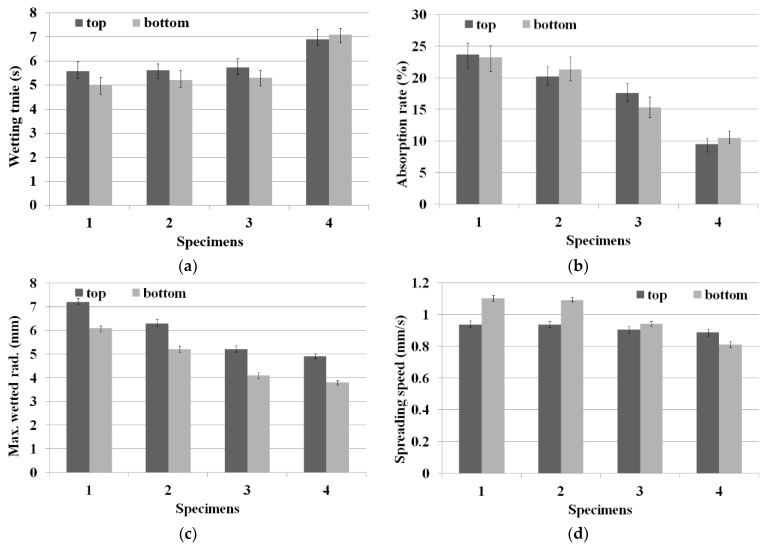
Diagram of MMT results of the fabric specimens ((**a**) wetting time, (**b**) absorption rate, (**c**) max.wetted.rad, and (**d**) spreading speed).

In this study, the MMT experiment was carried out to evaluate the moisture absorption and rapid drying properties of the fabric specimens. Table 6 lists the moisture absorption and rapid drying properties of the four types of fabric specimens measured using the MMT method. Figure 7 presents diagrams of the wetting time, absorption rate, maximum wetted radius, and spreading speed of the fabric specimens. The deviations in Figure 7 denote the difference between the maximum and minimum values of the experimental data. ANOVA (F-test) was performed to verify the statistical significance of the experimental data with a 99% confidence limit (1% of significance level). Table 7 lists the ANOVA results of the four moisture absorption and drying properties between each fabric specimen. The mean wetting time, absorption rate, maximum (max.) wetted radius (rad.), and spreading speed were statistically significant for each fabric specimen examined, as F_0_ (V/Ve) > F (3, 16, 0.99) and *p* < 0.01.

The wetting time of the Al_2_O_3_/ATO-embedded fabrics (specimens 1, 2, and 3) was shorter than that of the regular PET fabric (specimen 4), which means that the moisture absorption property of the Al_2_O_3_/ATO-embedded fabrics is superior to that of the regular PET fabric (Figure 7a). This was attributed to the rapid drying characteristics of the absorbed moisture in the yarn by the heat due to more FIR being radiated from the Al_2_O_3_/ATO particles than that from TiO_2_ particles in the yarns, which was previously verified by the higher FIR emissivity of the Al_2_O_3_/ATO-embedded yarns (specimens 1, 2, and 3) in Table 4, i.e., a rapid drying enables absorbed moisture from human skin to accelerate the capillary wicking into the horizontal and vertical directions in the fabrics, resulting in a shorter wetting time. This result was consistent with previous findings [5,8,11], reporting that ZrC- and Al_2_O_3_-embedded fabrics showed more rapid drying properties than the regular PET fabric. The wetting time of the specimen 1 (50 wt. %) was shorter than that of specimens 2 (60 wt. %) and 3 (70 wt. %), which was attributed to the rapid drying property caused by the lesser heat-shielding effect of specimen 1 compared with specimens 2 and 3. This observation was explained by the higher emissivity of yarn specimen 1 than that of specimens 2 and 3 in Table 4, i.e., fewer ATO particles in the core of specimen 1 make it possible to shield less heat radiated from the Al_2_O_3_ particles embedded in the core, resulting in rapid drying and a shorter wetting time.

The absorption rate of the Al_2_O_3_/ATO-embedded fabrics (specimens 1, 2, and 3) was higher than that of regular PET fabric (specimen 4), which was also attributed to the rapid drying property by heat due to more FIR being radiated from the Al_2_O_3_/ATO particles in the yarns, as shown in Figure 7b. In addition, the absorption rate of specimen 1 was higher than specimens 2 and 3, which means that the moisture absorption of the fabric with a low core wt. % of Al_2_O_3_/ATO (sheath/core: 5/5) is superior to that of the fabrics with high ones (sheath/core: 4/6 and 3/7). This can also be explained by the heat-shielding effect of the ATO particles, i.e., the high wt. % ratio (70 % (3:7)) of the core in the Al_2_O_3_/ATO-embedded yarns provides a higher amount of ATO in the yarns, making it a less effective heat release due to the lower emissivity than the low wt. % ratio yarn (50% (5:5)) because of the heat-shielding property of the ATO particles, as mentioned previously (Table 4).

Figure 7c presents the maximum wetted radius that affects the drying time of the fabric specimens. The maximum wetted radius of the Al_2_O_3_/ATO-embedded fabrics (specimens 1, 2, and 3) was larger than that of the regular PET fabric (specimen 4), suggesting that the moisture dropped in the MMT experimental apparatus is absorbed and dried quickly by the greater amount of heat radiated from the Al_2_O_3_/ATO particles in the yarn, and then penetrates rapidly into the fabrics, resulting in a short wetting time and an increased absorption rate and maximum wetted radius. The current findings are in accordance with Furuta et al. [5], Bahng et al. [8], and Kim et al. [11,12], who reported that ceramic-particle (ZrC, Al_2_O_3_, and SiO_2_)-embedded fabrics exhibited superior water absorption and drying properties compared to those of the regular PET fabric. In addition, the maximum wetted radius of fabric specimen 1 (sheath/core: 5:5) was larger than that of fabric specimens 2 and 3 (sheath/core: 4:6 and 3:7), which was attributed to the higher effectiveness for heat release by the higher emissivity of the yarn specimen 1 (Table 4) than that of yarn specimens 2 and 3, which was also caused by the higher heat-shielding property due to the greater number of ATO particles in the high-core-ratio yarns, as mentioned previously.

As shown in Figure 7d, the spreading speed of the Al_2_O_3_/ATO-embedded fabrics (specimens 1, 2, and 3) was faster than that of the regular PET fabric (specimen 4), which was attributed to the superior drying property due to higher heat release by the higher emissivity (Table 4) of the Al_2_O_3_/ATO-embedded yarns. The spreading speed of fabric specimen 1 was faster than that of fabric specimens 2 and 3. This result can also can be explained similarly to that mentioned in the maximum wetted radius. These MMT results suggest that the Al_2_O_3_/ATO-embedded fabric with a low core ratio in the sheath/core yarn has superior moisture absorption and rapid drying properties compared to the fabric with a high core ratio, giving it a comfortable wear feeling while wearing the clothing.

### 3.4. Thermal Characteristics of Al_2_O_3_/ATO-Embedded Fabrics

The thermal properties such as the heat retention rate (I), thermal conductivity (K), and moisture vapor resistance (R_et_) were measured using the KES-F7 (Kato Tech Co., Japan) and a sweating-guarded hot plate system (Therm DAC, UK) and compared with the wt. % of Al_2_O_3_/ATO particles in the Al_2_O_3_/ATO-embedded yarns. Table 8 lists the experimental results.

**Figure 8:**
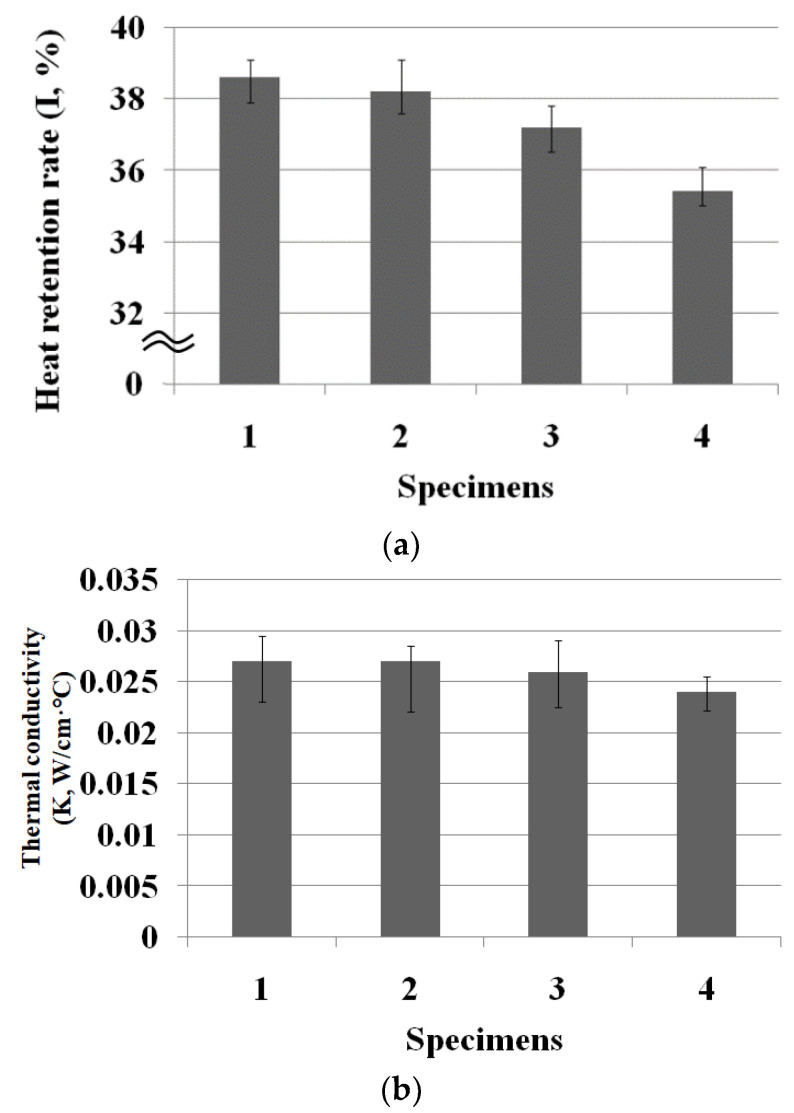
Diagram of the dry thermal properties of the four fabric specimens ((**a**) heat retention rate and (**b**) thermal conductivity).

Figure 8 presents a diagram of the dry thermal properties of the Al_2_O_3_/ATO-embedded fabrics. The deviation in the Figure 8 indicates the difference between the maximum and minimum values of the experimental data. ANOVA (F-test) was carried out to verify the statistical significance of the experimental data with a 95% confidence limit (5% of significance level). Table 9 lists the ANOVA data for the dry and wet thermal properties of the four fabric specimens. The mean values among the four fabric specimens for I, K and R_et_ were statistically significant, respectively, as F_0_ (V/Ve) > F (3, 16, 0.95) and *p* < 0.05. As shown in Figure 8a, the heat retention rates of the Al_2_O_3_/ATO-embedded fabrics (specimens 1, 2, and 3) were higher than that of the regular PET fabric (specimen 4), respectively. This was attributed to more heat released from the absorption or accumulation of the higher FIR radiation emitted from the Al_2_O_3_/ATO particles in the yarns, which was previously verified by the higher emissivity caused by the FIR radiation emitted from the Al_2_O_3_/ATO particles in the yarn specimens 1, 2, and 3 (Table 4). In addition, the heat retention rate of fabric specimen 1 was higher than that of fabric specimens 2 and 3 because of the inferior heat-shielding effect of the ATO particles in the low-core-ratio yarn specimen 1, as explained previously in Table 4. These findings suggest that low-core-ratio (50%) Al_2_O_3_/ATO-embedded fabric imparts superior thermal wear comfort compared to the high-core-ratio (60 and 70%)-included fabrics while the wearing clothing, resulting in a comfortable, warm feeling.

As shown in Figure 8b, the thermal conductivities (K) of the Al_2_O_3_/ATO-embedded fabrics (specimens 1, 2 and 3) were higher than that of the regular PET fabric (specimen 4), which was attributed to the higher thermal conductivities of the Al_2_O_3_ and ATO in specimens 1, 2, and 3 than that of the TiO_2_ embedded in the specimen 4 [41]. In addition, the thermal conductivity of specimen 1 was slightly higher than that of specimens 2 and 3, which was caused by the heat-shielding property of the ATO particles embedded in the yarn, resulting in a higher thermal conductivity of the low-core-ratio fabric (50%, specimen 1) than the high-core-ratio fabrics (60% and 70%, specimens 2 and 3, respectively). This means that a higher ATO-embedded fabric has lower thermal conductivity because ATO absorbs and shields FIR radiation and heat energy, as reported elsewhere [28,42].

### 3.5. Moisture Vapor Resistance of the Al_2_O_3_/ATO-Embedded Fabrics

The moisture vapor resistance (i.e., breathability) of the Al_2_O_3_/ATO-embedded fabrics is a very important property to evaluate the wear comfort of the clothing. The moisture vapor resistance (R_et_) by the sweating-guarded hot plate method was measured using the wet thermal transmittance of the moisture vapor, as explained in Equation (5).

**Figure 9:**
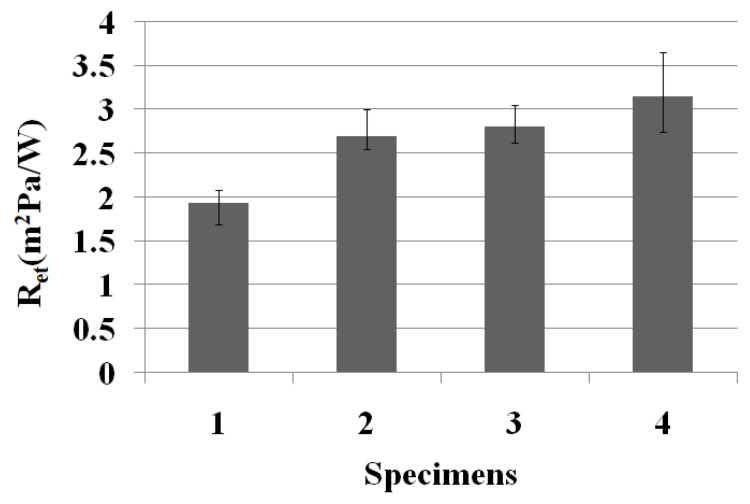
Moisture vapor resistance of the four types of fabric specimen.

Figure 9 presents the moisture vapor resistance of the four types of fabric specimens. As shown in Figure 9, the moisture vapor resistance of the Al_2_O_3_/ATO-embedded fabrics (specimens 1, 2, and 3) was lower than that of the regular PET fabric (specimen 4), i.e., they exhibited better breathability. This was attributed to the more heat particles by higher FIR radiation emitted from the Al_2_O_3_/ATO-embedded yarns (Table 4), which accelerates moisture vapor evaporated from the human skin, enabling it to push out and to escape easily from the human body and fabric, lowering the moisture vapor resistance. This result was consistent with previous findings [5,8,11] obtained using different ceramic-particle-embedded fabrics. According to their findings with the ZrC- [5,11] and Al_2_O_3_- [8] embedded fabrics, ceramic-embedded fabrics exhibited superior moisture vapor transmittance than the regular PET fabric.

Regarding the moisture vapor resistance according to the wt. % of the sheath/core of the Al_2_O_3_/ATO-embedded yarn, the moisture vapor resistance of fabric specimen 1 was lower than that of fabric specimens 2 and 3 (Figure 9), which was caused by the heat-shielding properties of the ATO ceramic particles, i.e., more ATO particles in the yarns by the higher wt. % of the ATO particles in fabric specimens 2 and 3 enhance the heat shielding and insulation, resulting in lower heat release with lower FIR emissivity of the yarn specimens 2 and 3, which hinders and decelerates the moisture vapor movement as mentioned above, resulting in higher moisture vapor resistance of fabric specimens 2 and 3. This finding suggests that the fabric with a low core ratio of Al_2_O_3_/ATO (50 wt. %, specimen 1) imparts more heat release than the high-core-ratio fabric (70 wt. %, specimen 3), resulting in easy moisture vapor permeability (i.e., low moisture vapor resistance/good breathability). This provides a more comfortable feeling while wearing the clothing.

## 4. Conclusions

In this study, the wear comfort of Al_2_O_3_/ATO-embedded fabrics with different sheath/core ratios of the constituent yarns was examined in relation to the FIR emissivity characteristics of Al_2_O_3_/ATO particles and the heat-shielding property of ATO particles embedded in the yarns. The heat shielding effect of the ATO particles in the highly Al_2_O_3_/ATO-embedded yarns enabled it to shield the FIR emitted from sunlight, resulting in the lower emissivity and emissive power of the highly Al_2_O_3_/ATO-embedded yarn than the lower embedded yarn. Accordingly, the lower Al_2_O_3_/ATO-embedded yarns are required to obtain excellent thermal radiation of the fabrics. The maximum surface temperature of the Al_2_O_3_/ATO-embedded fabric with a low core ratio (50 wt. %) was higher than that of the fabric with a high core ratio (70 wt. %). This result was verified by the higher emissivity and emissive power caused by the higher FIR radiation of the Al_2_O_3_/ATO-embedded yarn with a low core ratio (50 wt. %), which was attributed to the heat-absorbing and -shielding properties of the ATO particles in the yarns.

The heat shielding and release properties of the Al_2_O_3_/ATO-embedded fabrics according to the wt. % of Al_2_O_3_/ATO particles embedded in the yarns affected the wear comfort properties such as the moisture absorption and rapid drying properties, heat retention rate, and moisture vapor resistance, which are summarized as follows. The Al_2_O_3_/ATO-embedded fabric with a low core ratio exhibited superior moisture absorption and rapid drying properties to the fabric with a high core ratio, giving it a comfortable feel to wear. The Al_2_O_3_/ATO-embedded fabric with a low core ratio exhibited a higher heat retention rate than the fabric with a high core ratio as illustrated by the greater heat release because of the inferior heat-shielding effects by the fewer ATO particles in the yarn, suggesting that the low-core-ratio yarn is suitable for warm-up fabric with UV-cut and anti-static properties because of its superior thermal wear comfort with a warm feeling.

In addition, the fabric with a low core ratio showed a lower moisture vapor resistance than that of the high-core-ratio fabric, which was caused by higher heat release with higher FIR emissivity of the low-core-ratio fabric because of less heat-absorbing and -shielding effects of the ATO particles in the low-core-ratio yarns, resulting in easy moisture vapor permeability (good breathability) and giving it a comfortable feel while wearing the clothing. Hence, Al_2_O_3_/ATO-embedded fabric with a low core ratio is of practical application for multi-functional fabrics with good absorption and moisture vapor permeability and thermal wear-comfort feeling through lower inclusion of the ATO particles in the core of the sheath/core PET yarns. In addition, further research on practical use and application as a new material with protective properties is needed for application of cold-weather protective clothing with Al_2_O_3_/ATO-embedded fabric materials.

## Figures and Tables

**Table 1:** M/B chips mixed with PET polymer and ceramic particles.

	M/B Chip	Al_2_O_3_ Chips	ATO Chips	PET Chips (TiO_2_)
Characteristics				
Concentration (wt. %)		20	20	0.36
Average size (nm) of ceramic particles		869	200–300	400–500
M/B shape (chip)		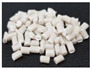	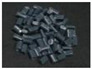	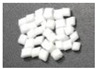
SEM images of the ceramic particles		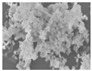	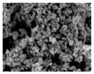	Specification I.V.: 0.665 Tg: 80.7 °C Tm: 252 °C

Note: I.V., intrinsic viscosity; Tg, glass transition temperature; Tm, melting temperature.

**Table 2:** Mixing ratio of M/B and yarn specimen spun in a conjugated melt spinning machine.

	PET Chip Weight (kg)	M/B Chip Weight (kg)		Mixed Polymer Weight (kg)	Mixing Ratio of M/B (wt. %)		
		Al_2_O_3_ATO			Al_2_O_3_	ATO	Total
Al_2_O_3_/ATO embedded PET M/B	47.25	2	0.75	50	0.8	0.3	1.1
Al_2_O_3_/ATO embedded PET yarn specimen	Yarn specimen 1	Yarn specimen 2		Yarn specimen 3	Yarn specimen 4		
	Sheath/core ratio (5:5)	Sheath/core ratio (4:6)		Sheath/core ratio (3:7)	Regular PET		

**Table 3:** Specification of the four types of fabric specimens.

Fabric Specimen No.	Yarn Count (d/f)		Sheath: Core (S/C) Ratio	Fabric Density (Picks, Ends/in)		Thickness	Weight	Weave Structure
	Wp	Wf		Wp	Wf	(mm)	(g/m^2^)	
1	PET 50/72	Al_2_O_3_/ATO S/C PET 50/24	5:5	154	100	0.13	85.4	plain
2	PET 50/72		4:6	154	100	0.12	84.8	
3	PET 50/72		3:7	154	100	0.13	85.6	
4	PET 50/72	Reg PET 50/72	-	154	100	0.12	84.2	

**Table 4:** Emissivity and emissive power of the four types of yarn specimens.

Specimen	Specimen 1		Specimen 2		Specimen 3		Specimen 4	
	Mean	Dev.	Mean	Dev.	Mean	Dev.	Mean	Dev.
Emissivity	0.885	0.1 × 10^−3^	0.884	0.2 × 10^−3^	0.882	0.1 × 10^−3^	0.866	0.2 × 10^−3^
Emissive power (W/m^2^·μm)	3.44 × 10^2^	0.001 × 10^2^	3.43 × 10^2^	0.002 × 10^2^	3.42 × 10^2^	0.001 × 10^2^	3.40 × 10^2^	0.001 × 10^2^

Note: dev = max − min.

**Table 5:** ANOVA of the emissivity and emissive power.

	F-Value (F_0_)	F (3, 16, 0.95)	*p*-Value
Emissivity	12.02	3.239	2.27 × 10^−4^
Emissive power	3091.65	3.239	2.58 × 10^−22^

Note: F_0_ (V/Ve), test statistic; V, mean square; Ve, error variance; F (3, 16, 0.95), critical value.

**Table 6:** Absorption and drying properties of the four types of fabric specimens by the MMT experiment.

Specimens No.		Wetting Time (s)		Absorption Rate (%)		Max.Wetted Rad.(mm)		Spreading Speed (mm/s)	
		Top	Bottom	Top	Bottom	Top	Bottom	Top	Bottom
1	Al_2_O_3_/ATO S/C 5:5	5.580	5.011	23.635	23.20	7.2	6.1	0.9362	1.102
2	Al_2_O_3_/ATO S/C 4:6	5.625	5.213	20.204	21.30	6.3	5.2	0.9360	1.091
3	Al_2_O_3_/ATO S/C 3:7	5.737	5.312	17.622	15.30	5.2	4.1	0.9040	0.942
4	Regular PET fabric	6.912	7.102	9.504	10.50	4.9	3.8	0.8880	0.810

**Table 7:** ANOVA results for absorption and drying properties of the fabric specimens.

Absorption and Drying Properties		F-Value (F_0_)	F (3, 16, 0.99)	*p*-Value
Wetting time (s)	top	29.50	5.292	9.38 × 10^−7^
	bottom	70.36	5.292	1.96 × 10^−9^
Absorption rate (%)	top	130.80	5.292	1.82 × 10^−11^
	bottom	93.69	5.292	2.31 × 10^−10^
Max.wetted rad. (mm)	top	380.55	5.292	4.42 × 10^−15^
	bottom	613.86	5.292	1.01 × 10^−16^
Spreading speed (mm/s)	top	11.91	5.292	2.38 × 10^−4^
	bottom	543.26	5.292	2.65 × 10^−16^

**Table 8:** Thermal properties of the fabric specimens.

Specimens/Properties		Specimen 1	Specimen 2	Specimen 3	Specimen 4
		Al_2_O_3_/ATO S/C 5:5	Al_2_O_3_/ATO S/C 4:6	Al_2_O_3_/ATO S/C 3:7	Regular PET
		Mean	Mean	Mean	Mean
Dry thermal properties	I (%)	38.60	38.20	37.20	35.40
	K (W/cm °C)	0.0278	0.0271	0.0264	0.0245
Wet thermal property	R_et_(m^2^ Pa/W)	1.94	2.70	2.81	3.15

**Table 9:** ANOVA data for the dry and wet thermal properties of the fabric specimens.

Dry and Wet Thermal Properties	F-Value (F_0_)	F (3, 16, 0.95)	*p*-Value
I (%)	43.13	3.239	6.82 × 10^−8^
K (W/cm °C)	4.01	3.239	0.0263
R_et_(m^2^ Pa/W)	45.19	3.239	4.90 × 10^−8^

## Data Availability

The data presented in this study are available on request from the corresponding author.
